# Ectopic pregnancy secondary to in vitro fertilisation-embryo transfer: pathogenic mechanisms and management strategies

**DOI:** 10.1186/s12958-015-0025-0

**Published:** 2015-04-12

**Authors:** Bassem Refaat, Elizabeth Dalton, William L Ledger

**Affiliations:** Laboratory Medicine Department, Faculty of Applied Medical Sciences, Umm Al-Qura University, Al-Abdiyah Campus, PO Box 7607, Makkah, KSA; School of Women’s & Children’s Health, University of New South Wales, Sydney, NSW 2031 Australia

**Keywords:** Ectopic pregnancy, Assisted conception, Infertility, Fallopian tube, Clinical management

## Abstract

**Background:**

Ectopic pregnancy (EP) is the leading cause of maternal morbidity and mortality during the first trimester and the incidence increases dramatically with in vitro fertilisation and embryo transfer (IVF-ET). The co-existence of an EP with a viable intrauterine pregnancy (IUP) is known as heterotopic pregnancy (HP) affecting about 1% of patients during assisted conception. EP/HP can cause significant morbidity and occasional mortality and represent diagnostic and therapeutic challenges, particularly during fertility treatment. Many risk factors related to IVF-ET techniques and the cause of infertility have been documented. The combination of transvaginal ultrasound (TVS) and serum human chorionic gonadotrophin (hCG) is the most reliable diagnostic tool, with early diagnosis of EP/HP permitting conservative management. This review describes the risk factors, diagnostic modalities and treatment approaches of EP/HP during IVF-ET and also their impact on subsequent fertility treatment.

**Methods:**

The scientific literature was searched for studies investigating EP/HP during IVF-ET. Publications in English and within the past 6 years were mostly selected.

**Results:**

A history of tubal infertility, pelvic inflammatory disease and specific aspects of embryo transfer technique are the most significant risk factors for later EP. Early measurement of serum hCG and performance of TVS by an expert operator as early as gestational week 5 can identify cases of possible EP. These women should be closely monitored with repeated ultrasound and hCG measurement until a diagnosis is reached. Treatment must be customised to the clinical condition and future fertility requirements of the patient. In cases of HP, the viable IUP can be preserved in the majority of cases but requires early detection of HP. No apparent negative impact of the different treatment approaches for EP/HP on subsequent IVF-ET, except for risk of recurrence.

**Conclusions:**

EP/HP are tragic events in a couple’s reproductive life, and the earlier the diagnosis the better the prognosis. Due to the increase incidence following IVF-ET, there is a compelling need to develop a diagnostic biomarker/algorithm that can predict pregnancy outcome with high sensitivity and specificity before IVF-ET to prevent and/or properly manage those who are at higher risk of EP/HP.

## Background

**Figure 1:**
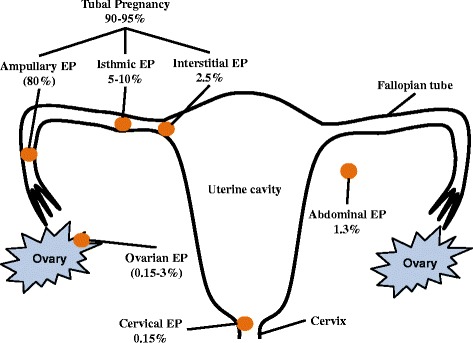
Sites and prevalence of ectopic pregnancy following IVF-ET.

Ectopic pregnancy (EP) is a form of abnormal pregnancy in which the fertilised ovum implants outside the intrauterine cavity, with the ampullary region of the Fallopian tube being the most common site of implantation (Figure 1) [1]. EP represents 1-2% of all pregnancies and haemorrhage from an EP due to tubal rupture remains the most common cause of maternal mortality in the first trimester of pregnancy [2].

Infertility is a problem affecting 8-12% of couples worldwide [3]. The associations between infertility and EP are complex, as one of them could be simultaneously a cause and the other a consequence [4,5]. There is an increased risk of developing EP following fertility treatment, which could be due to the effects of the treatment or the pre-existing disorder [6].

Since the birth of the first successful in vitro fertilisation (IVF) baby in 1978 [7], there has been an increase demand for assisted reproductive technologies (ART), including intrauterine insemination and IVF-embryo transfer (IVF-ET) with or without intracytoplasmic sperm injection [8]. Nevertheless, IVF-ET is a major risk for the development of EP and the incidence is greater by 2–3 folds than that seen in the general population. IVF may also result in a heterotopic pregnancy (HP), which is an EP together with a viable intrauterine pregnancy (IUP) [6].

Several factors related to the cause of female infertility and applied technical procedures during IVF-ET have been described as major risk factors for EP/HP. The current review discusses these risk factors, available diagnostic modalities and management approaches for the treatment of EP/HP following IVF-ET treatment, and the impact of these abnormal pregnancies and their treatment on the chances of success of subsequent fertility treatment.

## Methods

‘Medline’ and ‘EMBASE’ were searched using the terms ‘in vitro fertilisation’, ‘embryo transfer’, ‘controlled ovarian stimulation’, ‘risk factors’, ‘diagnosis’, ‘ultrasound’, ‘human chorionic gonadotropin’, ‘laparotomy’, ‘laparoscopy’, ‘salpingectomy’, ‘salpingostomy’, ‘methotrexate’, ‘potassium chloride’, ‘hyperosmolar glucose’, ‘vasopressin’, ‘embryo reduction’, ‘ovarian reserve’ and ‘pregnancy rate’ in combination with ‘ectopic pregnancy’, ‘heterotopic pregnancy’, ‘tubal pregnancy’, ‘interstitial pregnancy’, ‘ovarian pregnancy’, ‘caesarean scar pregnancy’, ‘cervical pregnancy’ or ‘abdominal pregnancy’ for studies published between 2004 and 2014.

Publications in English and within the past 6 years were selected, but commonly referenced and important older publications were not exclude. The reference lists of articles identified by this search strategy were also searched and those judged as relevant were also included. For a study to be included, it needed to be focused on incidence, diagnosis, clinical management and effect on subsequent IVF cycle of EP/HP during IVF-ET treatment. Studies that were solely focusing on EP following spontaneous conception were not included except of the management of HP due to its infrequency.

## Results

### Prevalence

EP is estimated to be 1-2% of all natural conceptions and the incidence increases following ART [4]. The prevalence of EP following ART ranges between 2.1 to 8.6% of all pregnancies and it can reach up to 11% in female patients with a history of tubal factor infertility [9]. Death from EP has been reported to represent 5% and 10% of all maternal deaths in developed and developing countries, respectively [2].

Spontaneous HP was considered to be very rare with an incidence of 1 in 30,000 pregnancies. The incidence of HP has also increased following ART and it has been reported that it complicates about 0.8% of pregnancies following infertility treatment [10].

### Pathogenic mechanisms

**Figure 2:**
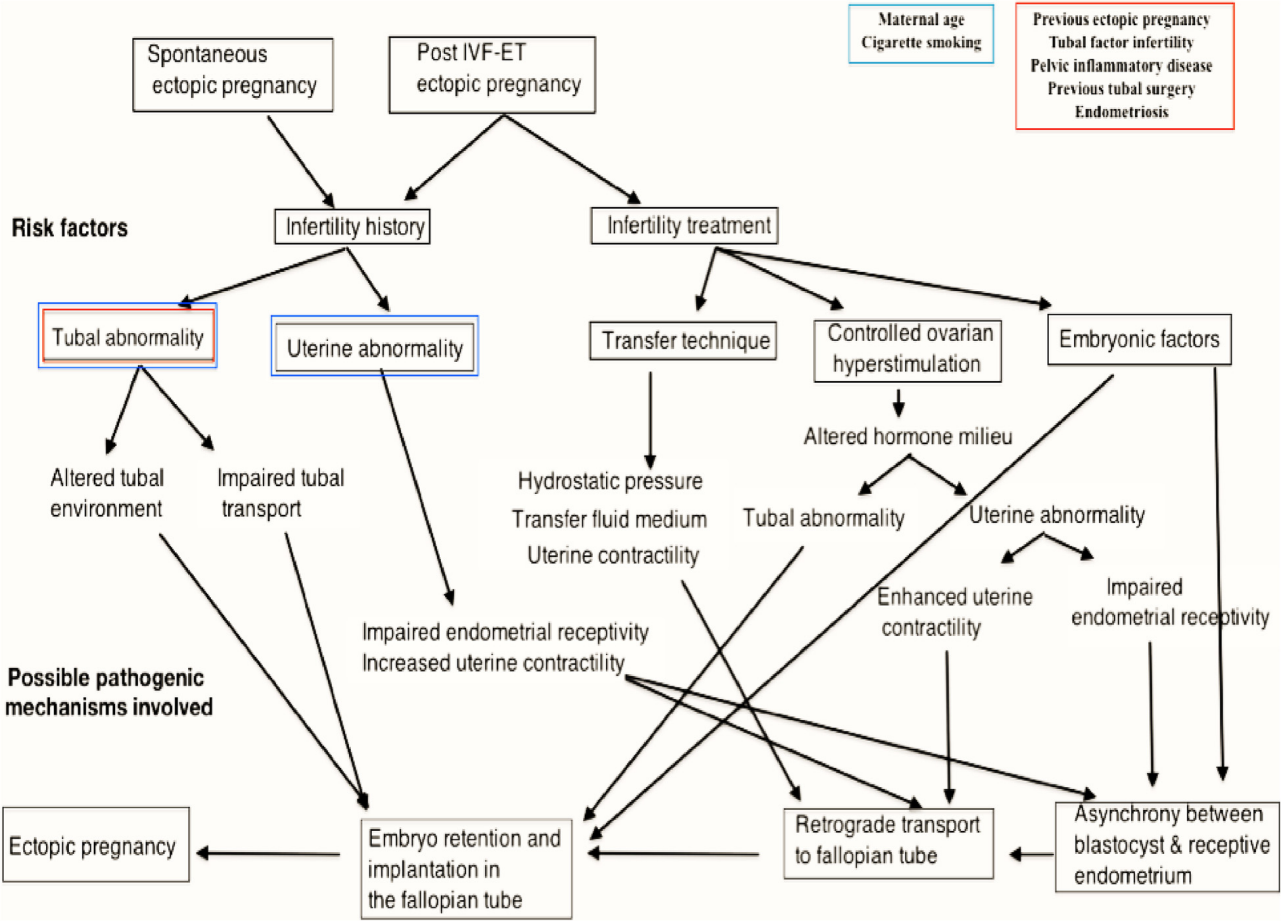
Pathogenic mechanisms. Potential mechanisms involved in the pathogenesis of tubal pregnancy after natural and IVF conception, in relation to established risk factors.

Tubal pregnancies that occurring naturally and following IVF-ET share the same tubal risk factors, suggesting that tubal damage has a predominant role in the pathogenesis of both [6]. The proposed pathogenic mechanisms associated with risk factors for EP either following natural or assisted conception are summarized in Figure 2.

Our understanding of the pathophysiology of EP is limited [11,12]. The current literature supports the hypothesis that the major cause of tubal implantation is malfunction of the tube itself, although embryonic and uterine factors may also be implicated. Tubal malfunction results from alterations in tubal transport mechanisms and expression of molecules that normally inhibit blastocyst implantation in the Fallopian tube [13-15].

However, in the case of EP post IVF-ET, in which passage of the embryo along the Fallopian tube does not occur, additional factors that prevent intrauterine implantation must precede ectopic implantation of the embryo. Differentiating between the mechanisms involved in natural and post IVF-ET tubal pregnancy is difficult. To our knowledge, only one study has compared tubal pathology in natural and IVF ectopic pregnancies, using E-cadherin as a marker of implantation potential [16]. Further biological studies using this comparative approach are necessary in order to elucidate the mechanisms involved.

Another explanation for EP during IVF-ET would be impairment of tubal function and endometrial receptivity with ectopic implantation occurring following failure of the normal biological interactions between endometrium, Fallopian tube and embryo due to controlled ovarian stimulation (COS) and the subsequent alteration in hormonal milieu [17,18]. Hence women with underlying tubal disease undertaking IVF may face a “double whammy” in risk of EP, due both to the tubal disease and adverse effects of superovulation on tubal function during the IVF cycle.

### Risk factors

Several factors increase the risk of EP following IVF-ET and they are associated either with the infertility history of the patient or specific factors related to IVF-ET techniques (Table 1).

**Table 1:** **Risk factors for EP during IVF-ET**

	**Maternal**	**IVF-ET Technique**
*Definite risks*	Tubal infertility	High volume of transfer media
	Pelvic inflammatory disease	Multiple embryo transfer
	History of tubal surgery	
	Previous ectopic pregnancy	
	Cigarette smoking	
	Endometriosis	
*Inconclusive risks*	Maternal age	Controlled ovarian stimulation
	Uterine abnormalities	Triggering oocyte maturation
		Luteal phase support
		In vitro fertilisation/maturation
		Assisted hatching
		Embryonic stage at transfer
		Fresh vs. Frozen embryo
		Transfer technique of embryo

Summary of definite and inconclusive risk factors for ectopic/heterotopic pregnancy during in vitro fertilisation and embryo transfer treatment.

#### Factors related to infertility history

The increase in the incidence of EP is associate with the use of ART. Even with a single embryo transfer, women undergoing ART treatment have a relative risk of 6.40 (95% CI: 4.38-9.35) of developing EP compared with natural conceptions [19]. The increase in EP has been associated with several maternal risk factors, mainly related to the woman’s infertility history.

#### Maternal age

Several research groups have shown that the risk of EP increases with advancing age, particularly in women over 35 years [20,21]. A steady increase was also reported in the incidence of EP with the increase in maternal age at conception from 1.4% at the age of 21 years to 6.9% in women aged 44 years or more [22]. An explanation for this trend with age could be the existence of a higher probability of exposure to most other risk factors with advancing age, increase in chromosomal abnormalities in trophoblastic tissue and age-related changes in tubal function [21].

Alternatively, other research groups have failed to detect an association between maternal age and the risk of developing EP [23-25], Further prospective studies with large numbers of participants are needed to determine whether a relation truly exists between maternal age and the risk of developing EP during IVF-ET.

#### Tubal factor infertility

Tubal factor infertility (TFI) is a major risk for EP when compared to other causes of infertility [6,26]. IVF-ET was originally designed to overcome TFI by placing the embryo(s) within the uterine cavity. However, it appears that the embryo, in some cases, can still enter the tube and establish an ectopic implantation [20].

TFI, compared with other causes of infertility, increases the risk of EP following IVF-ET with a prevalence of up to 11% of cases with TFI [9]. Similar results were also reported by a more recent study that examined the risk factors for EP following IVF in 712 women with an OR of 3.99 (95% CI: 1.23 to 12.98) for women with TFI compared with those with other infertility causes [23]. Another study has also described that the OR for EP in 900 women with planned pregnancy was 8.81 (95% CI: 1.68–21.21) for those with a history of TFI compared with 5.82 [95% CI: 3.47, 9.78] for non-tubal infertility. However, the authors have also reported that the adjusted odd ratio (AOR) was comparable between TFI (3.62; 95% CI: 1.52, 8.63) and non-TFI (3.34; 95% CI: 1.60, 6.93) [25].

#### Pelvic Inflammatory Disease

A history of pelvic inflammatory disease (PID) has also been implicated in the increased incidence of EP following either natural or assisted conception [26,27]. A past history of PID is associated with a 7.5 times greater risk of developing EP [28,29].

*Chlamydia trachomatis* infection is the most common sexually transmitted infection worldwide [30,31]. The immune response to this infection may lead to tubal occlusion, EP and infertility [27,32,33]. Despite spontaneous clearance of *C. trachomatis* infection, antibodies against the organism and its heat shock protein-60 (CHSP60) remain detectable for many years. The presence of these antibodies have been strongly associated with poor reproductive outcomes, including early pregnancy loss and EP following IVF [25,32].

The presence of IgA antibodies against *Chlamydia trachomatis* was associated with lower implantation and pregnancy rates among 235 patients undergoing IVF [34]. Live birth rate following IVF was also found to be lower in women with cervical anti-chlamydial and anti-CHSP60 IgA antibodies when compared with those who were negative for these antibodies [32]. Similar results were also observed for an effect on implantation rate in 253 IVF patients with follicular fluid anti-CHSP60 antibodies [35]. This is further supported by a more recent study that has shown that the risk of EP in 900 women with planned pregnancies and who had detectable *C. trachomatis* IgG was about five times of those with negative reaction (95% CI: 3.94–7. 04) [25].

In contrast, other studies have not supported a role for previous infection with *C. trachomatis* in the development of EP [23,36,37]. One longitudinal study found that there was no significant difference in pregnancy rates for women with or without chlamydial infection at baseline [38]. Similar results were also reported for 712 IVF patients [23]. The presence of antibodies against CHSP60 among 174 women with apparently normal Fallopian tubes at laparoscopy was also not associated with lower pregnancy rates during 3 years follow-up [39]. A recent review has therefore suggested that the inconsistency in the results could be related to flaws in study design and lack of a reliable method for measuring a history of pelvic infection [13]. The authors also argued that the current assumptions on the risks of adverse outcomes following pelvic infection in subsequent pregnancy are based on retrospective case–control studies, which are prone to many confounding variables [13].

#### Previous ectopic pregnancy

A history of EP is considered as a major risk factor for subsequent recurrence of EP and each successive occurrence significantly increases the risk [5,22]. The recurrence rate ranges from 15 to 20% in the case of one previous EP treated by linear salpingostomy, depending on the integrity of the contralateral tube and it increases to 32% in cases with two previous ectopic pregnancies. However, an intervening IUP lowers this rate [6,20,40].

A retrospective study measured the risk of EP following IVF in 181 women with a previous ectopic, with significantly higher risk of recurrence when compared with 377 women with other causes of infertility. The authors reported that the chance of developing EP was 45 times in the case group and the prevalence of EP was 8.95% compared with 0.75% in the control group [24]. Similar results with an OR of 9.68 (95% CI: 6.02-15.56) for were reported in a study of 900 EPs compared with 889 IUPs in women with planned pregnancy [25] and another study also demonstrated an OR of 57.93 (95% CI: 6.79-494.25) for developing EP in 150 women diagnosed with EP compared with 300 healthy pregnant women [21].

#### Previous tubal surgery

A history of tubal/pelvic surgery is another major risk factor for the development of EP following IVF, with the level of risk depending on the degree of damage and the extent of anatomic alteration [6,22]. Odds ratio for developing EP was 8.52 (95% CI: 5.91-12.27) for prior adnexal surgery, 11.02 (95% CI: 5.49, 22.15) for a previous tubal infertility surgery, 5.16 (95% CI: 1.25-21.21) for prior surgery for endometriosis and 17.70 (95% CI: 8.11-38.66) for a previous abdominal/pelvic surgery [21,23,25].

Hydrosalpynx is a main cause of tubal infertility and is also associated with a negative impact on the outcome of IVF treatment. Treatment of hydrosalpynx before IVF involves radical and conservative surgical approaches [41,42]. The rate of EP has been reported to be about 9% in patients undergoing IVF following tubal surgery for the treatment of hydrosalpinx [43,44].

#### Endometriosis

Endometriosis and its treatment has also been associated with the development of EP [6,25,45]. Endometriosis leads to the formation of pelvic and tubal adhesions, which could result in abnormal tubal function. Additionally, the Fallopian tubes could also be affected by other, less clearly understood effects of endometriosis [46,47], as well as by the medications that are administered to aid ovulation and improve fertility in patients with endometriosis [23].

#### Cigarette smoking

Cigarette smoking increases the risk of EP after natural conception by 1.6 to 3.5 times as compared to non-smokers, in a dose dependant manner [25,48,49]. Smokers also have a higher risk of developing EP following IVF treatment of about 3 times compared with non-smokers [24,25,49]. Laboratory studies in several species have shown that smoking decreases tubal and uterine motility [50-53].

#### Uterine abnormalities

Studies of the uterine factors that may be implicated in EP after IVF-ET have focused on the mechanism by which the embryo migrates into the Fallopian tube after intrauterine transfer. The possible effects of uterine abnormalities on implantation failure have not been well studied to date. A recent meta-analysis [54] concluded that “there is very little evidence that the established treatments improve outcomes, or that these pathologies have a negative effect on ART”. One study has reported that intramural fibroids are associated with a higher frequency of uterine peristalsis in the peri-implantation phase [55] but further work is required. The impact of uterine pathologies on risk of EP after IVF-ET remains to be elucidated.

#### Infertility treatment specific risk factors

An increased incidence and unusual forms of EP have been reported after IVF-ET and, in fact, the first pregnancy reported after IVF-ET was an EP [56]. Several studies have investigated the development of EP during IVF-ET and many technical issues of the treatment have been proposed as risk factors for EP. These risk factors (Table 1) include an altered hormonal milieu due to COS, the day and stage of embryo transfer, multiple embryo transfer, volume of transfer media and position of the catheter.

#### Controlled ovarian hyperstimulation

The alteration in the endocrine milieu in the stimulated/fresh cycle following COS may be a potential risk factor for the development of EP during IVF-ET. Possible mechanisms involve diminished endometrial receptivity [6], which could be supported by the observations that endometrial and subendometrial blood flow on the day of human chorionic gonadotropin (hCG) injection were significantly lower in IVF patients with miscarriage and EP when compared with those with viable IUP [57].

Higher circulating concentrations of oestradiol (E2) during stimulated cycles could also be associated with a reverse migratory process of the transferred embryo(s) [58]. A recent retrospective study showed that COS in patients with polycystic ovarian syndrome (PCOS) was associated with a greater risk of EP in fresh cycles (AOR 3.06; 95% CI: 1.34-6.96) but not with frozen-thawed embryo transfer (FTE) cycles. By further analysing the cases of EP in the non-PCOS patients, women with E2 levels > 4085 pg/mL had 2 times greater risk of developing EP (95% CI: 1.19-3.35). Hence, the authors suggested that the observed higher prevalence of EP during COS could be related to the hyperphysiologic levels of E2 associated with PCOS and/or ovulation induction [59].

Another review has suggested that the increased concentrations of progesterone in stimulated cycles could be beneficial in promoting endometrial implantation by decreasing uterine contractions when compared with FET cycles [6]. However, a later meta-analysis has identified a lack of randomised controlled trials (RCTs). The authors recommended the conduct of appropriately powered RCTs to compare natural cycle IVF with standard IVF, with outcomes including adverse events such as EP [60].

Available information on the possible effect of luteal phase support during the fresh cycle or for endometrial preparation in FET cycles in relation to the development of EP is conflicting. A retrospective study has shown that patients with high circulating concentrations of oestrogen and progesterone on the day of hCG injection had the highest frequency of EP compared with the low E2 and low progesterone groups [61]. Similar observations were also reported by other retrospective studies showing that programmed FET cycles were associated with higher rates of EP compared with natural cycles [62,63].

Conversely, a retrospective analysis of 1677 FTE cycles showed a non-significant difference in the prevalence of EP in natural versus programmed cycles [64]. This was supported by later data generated from an RCT and two retrospective studies that revealed no significant differences for use of progesterone in FET cycles in EP rates [65-67]. Several other multicentre RCTs have also measured the effect of different progesterone preparations for luteal phase support in fresh and FET cycles with no significant differences in the prevalence of EP [68-70]. Similar results were also reported by two recent meta-analyses including 91 RCTs of luteal phase support with progesterone or hCG [71,72].

In a prospective case–control study that included 100 IVF-ET cycles, a non-significant increasing trend in the prevalence of EP was observed between the use of GnRH-agonist in combination with hCG for triggering oocyte maturation compared with use of hCG alone [73]. A different retrospective study has described the opposite effect, with significantly higher rates of EP with GnRH-agonist trigger (5.3%) compared with recombinant hCG (1.4%) for oocyte maturation [74]. However, a recent Cochrane review has concluded that there is a lack of evidence that either treatment used for final oocyte maturation is associated with an increased risk of developing EP [75].

The association between COS and EP is still unclear and the results of the aforementioned studies are contradictory. The currently available data on the risk of developing EP following COS are mainly derived from retrospective studies or prospective case–control studies, which do not provide high quality scientific evidence for the suggested association between COS and EP. This has been reflected in the conclusions of several systematic reviews that have evaluated the different hormones during ART with the authors consistently stating that there is a lack of high quality evidence from RCTs that include the rate of EP in their primary or secondary objectives.

The recent concept of ‘individualised COS’ protocol that best fit the needs of each patient [76] makes the analysis of a possible association between COS and EP out with RCTs even more difficult. Future large multicentre RCTs with control for confounding variables are still needed to reach a solid conclusion on whether COS is a risk factor for the development of EP.

#### Embryonic factors

Assisted hatching (AH) has been proposed as a risk factor for developing EP after IVF [6]. In a previous retrospective analysis of 623 clinical pregnancies following IVF, a 5.4% rate of EP (14/258) was found in cases where AH was performed compared to 2.2% (8/365) in the non AH group [77]. However, a more recent RCT showed that AH neither improved pregnancy rate nor increased risk of EP in 121 women < 38 years [78] and a later retrospective analysis of 1126 AH cycles also reported that there was no effect on the prevalence of EP in an antibiotic and steroid treatment group compared with an untreated group [79]. It may be worthwhile to assess the effect of AH on the incidence of EP in a large multicentre study but recent evidence is against such an association.

Embryo transfer at the blastocyst stage appears to be the most biologically appropriate stage for intrauterine implantation as earlier stages of embryo development occur normally within the Fallopian tube [80]. Blastocyst transfer may also theoretically decrease the incidence of EP following IVF-ET as there may be decreased uterine contractility by day five after egg collection [81,82]. However, the potentially higher implantation rate per embryo at the blastocyst stage may increase the risk of EP compared with cleavage stage transfer [6] but a number of studies have indicated that, in practice, there is no difference in the incidence of EP between blastocyst and cleavage stage transfers [80,82,83]. EP rates have also been compared between frozen and fresh transfers of blastocyst with frozen transfer being shown to have the potential to decrease the frequency of EP compared with fresh transfer [62,66,84-86].

The ‘quality’ of the embryo may also contribute to risk of EP after IVF. Egg related factors such as chromosomal abnormalities may increase risk of EP [87] and, in a rodent study using an embryo donation model, superovulation with gonadotropins was suggested to impair blastocyst quality as well as endometrial receptivity [88]. A poor quality embryo may be less likely to undergo eutopic implantation resulting both in a decrease in the chances of establishment of an IUP and also increase in risk of EP.

Other possible embryonic factors that could induce EP following IVF include abnormal expression of adhesion molecules either by embryos fertilised in vitro or from tubal implantation sites following COS [16]. The abnormal expression has been attributed to the exposure of embryo(s) to different cytokines and growth factor milieu during in vitro culture compared with embryos conceived in vivo [13,16]. However, more sophisticated array studies are needed to explore possible altered expressions at the gene and protein levels by embryos following in vitro fertilisation and/or maturation.

#### Transfer technique

Another explanation for the development of EP following IVF-ET has been related to the induction of abnormal uterine contractions that may result in reverse migration of embryos from the uterine cavity into the Fallopian tube and ectopic implantation. Lesny et al. studied junctional zone uterine contractility during mock embryo-transfers and they reported that when the catheter was deliberately allowed to contact the uterine fundus, strong random contraction waves were observed in the fundal area and fluid was moved directly into the Fallopian tubes [89,90].

Another recent study has also demonstrated that uterine peristalsis increased significantly following 30 minutes of mock ET during the luteal phase of natural cycle in 112 infertile women. The fluid movement was positively and significantly correlated with the frequency of uterine peristalsis and the fluid was moved to the cervix, Fallopian tube and extruded in 5 (4.5%), 11 (9.8%) and 2 (1.8%) patients, respectively. The same research group later published another study on uterine peristalsis before ET in 292 infertile women undergoing fresh and FET cycles. Consistently, there was a significant negative correlation between uterine contractions and the achievement of clinical pregnancy. Moreover, uterine peristalsis > 2 waves/minute was a major risk factor for not achieving clinical pregnancy (OR 0.49; 95% CI: 0.34-0.70). The authors have therefore suggested that high uterine peristalsis could adversely affect embryo implantation and lead to low implantation rate and/or EP [91,92].

The technique of ET could also influence the rate of ectopic implantation due to forcing the embryo through tubal ostia by hydrostatic pressure or by using large volume of transfer medium [93-95]. Marcus et al. observed that patients having EP received a higher volume of culture medium than those having normal IUP [96]. Knutzen et al. also showed that after injecting 50 μl of radio-opaque fluid through a standard ET catheter, the material was passed either totally or partially into the Fallopian tubes in 44% of patients, suggesting that the chance of the embryo being carried into the tube immediately after high volume transfer [97].

The position of transfer catheter and the distance from the fundal endometrium to either the tip of catheter or to an air bubble within the catheter have also been investigated as potential risk factors for low pregnancy rate and development of EP. An RCT showed that IUP following deep fundal transfer was 12.4% per cycle with a 1.5% EP versus 14.2% IUPs per cycle with a 0.4% ectopic rate after midfundal transfer. The midfundal technique was suggested to be superior because of a lower risk of EP without any reduction in IUP rate [94,95]. This has been further supported by Coroleu et al. (2002) who reported that the placement of the transfer catheter close to the fundus endometrial surface (1 cm) resulted in EP while at a distance of 15–20 millimetres from the fundus achieved higher implantation and pregnancy rates [98].

Others have recommended performing ET in a position in which the fundus is at the highest point above the horizon and to perform the transfer slowly over at least 10 seconds [99]. They also suggest that placement of the catheter tip near the fundus appeared to transfer the embryos into the tube when transfer was performed more quickly, possibly resulting in EP and that transfer of embryos to a standard midcavity position results in a lower EP rate [99].

However, recent reports have also suggested that an optimal transfer location of a distance < 10 mm and > 5 mm from the fundal endometrium results in higher pregnancy rates compared with groups where the tip of the transfer catheter was placed further from the fundus. There were no cases of EP [100-102].

Multiple embryo transfer has always been associated with increased risk of EP with transfer of two or less embryos carrying lower risk than after three or more [103]. The current guidelines on the number of embryos to transfer, which are based on the maternal age, could therefore decrease the incidence of EP following IVF especially in younger patients with a single embryo transfer [103,104]. Remarkably, a single elective embryo transfer is associated with higher risk of EP (RR 6.40; 95% CI: 4.38-9.35) compared with spontaneously conceived singletons as reported by a recent meta-analysis [19]. Furthermore, a recent study by Chai et al. (2014) has shown that there was no significant difference in the frequency of EP between 74 women with single embryo transfer (2.5%) when compared with 132 women with double embryo transfer (5.2%) [105]. Nevertheless, single embryo transfer should be the preferred choice for all patients under 40 as it reduces risk of pregnancy complications, most notably premature birth with risk of subsequent impairment of the health of the offspring [106,107].

### Diagnosis of EP/HP following IVF-ET

EP/HP are serious complications during ART and present considerable diagnostic and therapeutic challenges. Adverse maternal outcomes may follow rupture of the EP with hypovolemic shock and blood transfusion requiring [10,14,108]. These events may also adversely affect the viable IUP in the case of HP. A single determination of serum hCG, even as early as 11–12 days after ET, and early transvaginal ultrasound (TVS) have been found to be predictive of pregnancy location [109,110] but cannot reliably identify a viable IUP with a co-existing EP.

#### Diagnostic modalities of EP

Serial quantitative measurement of hCG in combination with TVS is the currently accepted paradigm for clinical diagnosis and management of EP, with hCG monitoring being used to follow patients until complete resolution of the EP [4,5,111,112]. TVS can effectively detect small intrauterine and ectopic pregnancies when serum hCG level is 2000 IU/L [4,5,112] due to its superior resolution [111,113].

#### Human chorionic gonadotropin (hCG)

Although ART procedures and/or the pre-existing disorder may affect the course of hCG concentration [114], several studies on IVF patients have reported that a single serum β-hCG assay can accurately differentiate between viable and non-viable pregnancies. However, threshold values vary between the different studies, depending mainly on the exact day of the assay in relation to the date of ET.

Measuring serum concentrations of β-hCG in the peri-implantation period (day 5 post blastocyst transfer) was highly predictive of IVF-ET outcome and was able to differentiate ongoing pregnancies from failing pregnancies, including EP [115]. Another retrospective study showed that serum β-hCG on day 12 post ET for the diagnosis of EP had a sensitivity of 82.7%, specificity of 71.1%, positive predictive value (PPV) 15.5% and negative predictive value (NPV) 98.5% at a cut-off of 91 IU/L [110]. Similar results were also reported in 177 IVF cycles using 80 mIU/mL as the cut-off for a positive test result yielded a 94% sensitivity, 53.4% specificity and 80% positive predictive value for live birth [116]. Serum levels of β-hCG on day 15 following oocyte retrieval were shown to be dependent on the day of ET, being significantly higher after day 5 (198 ± 10.6 IU/L) compared with day 3 (103.6 ± 4.4 IU/L) transfers. The authors also reported that serum β-hCG above 78 IU/L and 160 IU/L were highly predictive of ongoing pregnancy for day 3 and day 5 transfers, respectively [117]. Another retrospective study showed that serum β-hCG at a cut-off value of 377.8 IU/L on day 17 post oocyte collection generated an area under the curve of 0.730 with 75.9% sensitivity and 61.2% specificity for the diagnosis of EP [118].

#### Ultrasound

Ultrasound can be used to locate a pregnancy anatomically and to see if the fetus is alive. In general, a gestational sac can be consistently identified by TVS at a cut-off level of serum β-hCG is 2000 IU/L, which is known as the discriminatory zone [119,120].

The finding of an empty uterine cavity can be associated with a small early pregnancy, EP or miscarriage. The correlation of the sonographic finding with serum level of hCG is therefore useful for achieving accurate diagnosis. If β-hCG level is below the discriminatory level, measurement of the hormone should be repeated every 48 hours with a repeat ultrasound when the level has reached the predetermined discriminatory level [121]. However, if the β-hCG is above the predetermined threshold or if it stabilises/fails to increase normally, then the diagnosis of an EP needs to be considered [121,122].

#### Diagnosis of HP

HP is a rare but potentially life-threatening event and early diagnosis and treatment are essential in order to best preserve the viable IUP and avoid maternal morbidity and mortality [122,123]. Hence this challenging condition should be suspected in all patients who conceive following ART regardless of the presence or absence of currently recognised risk factors, particularly after transfer of multiple embryos. Investigation of the adnexa by TVS is essential to exclude the possibility of HP. A moderate or large amount of free pelvic fluid should warrant particular care when assessing the adnexa with ultrasound, even in the presence of an IUP [124,125].

The diagnosis of HP is elusive and challenging due to the co-existence of the viable IUP which can generate an appropriate increase in the level of β-hCG. Thus the diagnostic value of hCG measurement is limited in cases with HP and the diagnosis is mainly dependent on the findings of TVS [122,124]. In a retrospective study that included a total of 184 HP following IVF-ET, 174 were diagnosed by TVS while 10 cases were missed. The three main types of ultrasonographic findings were: visualisation of extrauterine gestational sac in 57.3%, adnexal mass in 25.4% and ring sign in 17.2% of cases. The sensitivity and specificity of TVS in the diagnosis of HP was 92.4% and 100% respectively with positive and negative predictive values of 100% and 99.9%, respectively [126].

Most cases of HP are missed on initial presentation, which could be due a false sense of security provided by the sonographic finding of a viable IUP with failure to inspect the adnexa fully or the HP being too small to identify [122]. The amount of fluid in the cul-de-sac can assist in the diagnosis of HP. One study found that patients with abnormal cul-de-sac fluid were five times more likely to have HP as patients without it [127]. Causes of misdiagnosis include misidentification of an HP as a corpus luteal cyst [122,127] or due to mirror image artefact on TVS due to the patient having a full bladder during the process, which causes reflection of a viable IUP on a different part of the image and could be mistakenly diagnosed as HP [128].

A retrospective study investigated 28 EP/HP that ruptured due to misdiagnosis and/or delayed management and the main factors that led to delay were insufficient training in TVS and unawareness about EP/HP by the physicians, atypical symptoms and early rupture of EP. The authors suggested that TVS should therefore be performed at week 5 of gestation followed by serial measurement of serum β-hCG and repeated ultrasonography in suspicious patients [10].

### Treatment

EP is a major event in a woman’s reproductive life and is particularly tragic after a long and difficult course of treatment for infertility. Counselling and psychological support should also be given alongside clinical treatment if EP occurs, especially following infertility treatment [129].

EP/HP can be treated surgically, medically and occasionally by observation alone. Treatment must be customised to the clinical condition and future fertility requirements of the patient. However, the use of conservative approaches necessitates early diagnosis of EP/HP [130,131].

#### Surgical treatment

The traditional treatment for tubal pregnancy is laparotomy and salpingectomy. Laparoscopic approaches became more widely accepted after the development of video laparoscopy and the publication of the first series of successful use of laparoscopy for the treatment of EP in the 1980’s [132]. Laparoscopic surgery is the preferred approach in haemodynamically stable patients and has largely replaced the need for laparotomy due to improved postoperative recovery time and reduced morbidity [133,134].

Laparotomy is the preferred technique when the patient is haemodynamically unstable, if the surgeon has not been trained in laparoscopy or if laparoscopic surgery equipment is not available [133-135]. There is no difference in subsequent reproductive outcome between these surgical approaches. However, there is a trend towards higher rates of persistent trophoblast associated with laparoscopic surgery for EP [133-136].

Salpingectomy is preferable for tubal pregnancy in patients with uncontrolled bleeding, extensive tubal damage or recurrent EP in the same tube. Salpingostomy is indicated where the patient is haemodynamically stable, wishes to conserve her fertility, if there is an unruptured EP < 5 cm in diameter and, especially, when the contralateral tube is absent or damaged [134,137].

Two RCTs have compared salpingectomy and salpingostomy and have recently been published (DEMETER and ESEP). Both reported on reproductive outcomes after treatment of EP by both techniques [137,138]. Subsequent fertility, recurrent EP and IUP rates were similar following both approaches. However, persistent trophoblastic tissues were more common with salpingostomy with a relative risk of 15 (95% CI: 2.0-113.4) for persistence [137].

Hence, Mol et al. (2014) have suggested that salpingectomy should be the procedure of choice in women with tubal pregnancy and a healthy contralateral tube. These recommendations are also adapted by NICE in their guidelines for the management of EP [139]. Furthermore, a study that investigated the preference of patients regarding the type of operation found out that the majority of women preferred salpingectomy to avoid the possibility of another EP. However, the risk of persistent trophoblast was acceptable for these women if compensated by a small increase in the chances of an IUP following surgery [140].

#### Medical treatment

Medical management for EP requires effective early diagnosis as its success is inversely correlated with the level of serum hCG at diagnosis [130,141-143]. Several agents can be used for the treatment of EP, including methotrexate (MTX), potassium chloride (KCl) and hyperosmolar glucose [130,144,145]. The advantages of medical treatment are the avoidance of anaesthesia, surgery and its complications, preservation of tubal patency and function, and possibly cost effectiveness [130,145].

MTX, a cytotoxic drug that destroys rapidly dividing trophoblastic cells, is the most popular medical agent for the treatment of EP. MTX is a folic acid antagonist with metabolism and excretion in the liver and kidney, respectively [146,147]. MTX is given as single or multiple doses. The variable dose regimen, which consists of 4 injections, involves the addition of a reduced form of folate, citrovorum, to block the effect of MTX and to prevent adverse effects on other tissues [130,147]. Another treatment protocol with two doses of MTX and a schedule for follow-up for the patient similar to the ‘multiple doses’ regimen was first proposed in 2007. This protocol does not need the addition of citrovorum for the prevention of the drug side effects [143].

The dose of MTX is calculated according to body surface area (50 mg/m^2^) or body weight (1 mg/kg). For most women this will be between 75 and 90 mg [146,147]. MTX can be administered by intravenous or intramuscular injection, or by local injection under the guidance of either ultrasound or laparoscopy [141,147]. There is a significant risk of tubal rupture in unsuccessful cases following the use of MTX. Other side effects include abdominal pain due to tubal abortion, stomatitis and diarrhoea, hCG concentrations may rise for up to three days after MTX even in successful cases, and some may need a second dose of MTX [130,147].

Criteria for the use of MTX treatment in EP according to ACOG and NICE guidelines are listed in Table 2. Briefly, methotrexate can be used in haemodynamically stable patients with minimal or no symptoms and who have initial serum hCG concentrations < 5000 IU/L and EP size < 3.5 cm [130,139]. Patients should be advised to avoid sexual intercourse during treatment, becoming pregnant for 6 months post treatment and excessive exposure to sunlight and alcohol [147].

**Table 2:** **Criteria of methotrexate (MTX) treatment for ectopic pregnancy (EP)**

**MTX for EP**	
*Indications*	• Haemodynamically stable patients
	• Minimal or no symptoms
	• Serum hCG is < 5000 IU/L
	• Ectopic mass < 3.5 cm
	• No embryonic cardiac activity
	• Confirmed diagnosis of ectopic pregnancy
	• Able to comply with the follow-up
*Contraindications*	• Hemodynamically unstable
	• Suspected ruptured EP
	• Heterotopic pregnancy
	• Pregnancy of unknown location
	• Breastfeeding
	• Chronic liver disease
	• Renal disease
	• Active peptic ulcer or colitis
	• Active pulmonary disease
	• Immunodeficiency
	• Haematological disease
	• Sensitivity to MTX
	• Unable to comply with visits and follow-up

The treatment with MTX continues until hCG falls by 15% from its peak concentration within two days for single dose regimen or between day 4 and 7 for the multiple dose/two dose regimens [142,143,146,147]. A serum hCG measurement is performed on day 4 and 7 and a further dose is given if levels have failed to fall by more than 15% in the multiple dose/two doses regimens [142,143]. Approximately 50% of the treated patients will not require the full 4 doses in the ‘multiple dose’ protocol [130,147].

Several studies have compared laparoscopic salpingostomy with MTX, finding MTX to be almost as effective as surgery in terms of success rates and future fertility outcomes [135,136,138]. Tubal patency was documented by hysterosalpingography in 78% of cases and in 65% of patients who attempted to conceive again. Additionally, the incidence of recurrent EP was relatively low (12%) and was not significantly different from the observed rate (9%) with salpingostomy [138].

A meta-analysis reported that the success rate of MTX treatment was 92.7% and 88.1% for ‘multi-dose’ and ‘single dose’, respectively. The failure rate of ‘single dose’ protocol was estimated to be about 3 times higher than the ‘multiple dose’ regimen and the possibility of tubal rupture cannot be excluded even with falling hCG levels [148]. Signs of treatment failure or suspected rupture are indications to stop medical treatment and to shift to surgical management. Signs include haemodynamic instability, increasing abdominal pain regardless of trends in hCG levels, and rapidly increasing hCG concentrations (>53% over 2 days) after two doses or four doses in the ‘single’ and ‘multiple-dose’ regimens, respectively [138,143,149-151].

The use of MTX in women with a viable IUP is absolutely contraindicated as the drug would cause miscarriage or congenital malformations [130,152,153]. Hence, women with a pregnancy of unknown location (PUL) or HP following IVF-ET should be managed by other means [130].

Several other agents have been used for the medical treatment of EP. Sonographically guided local injection of KCl into the heart of the ectopic fetus can induce cardiac asystole with resolution of EP [154,155]. Hyperosmolar glucose can also be injected into the gestation sac causing local dehydration, necrosis of the trophoblastic tissue and resolution of EP [156,157]. These agents are not associated with fetal malformation but careful consideration should be given before use of hyperosmolar glucose since high doses could increase the risk of bleeding [158].

Recently, two studies have shown that the combination of gefitinib, an orally active epidermal growth factor receptor inhibitor, in combination with a single dose of intramuscular MTX (50 mg/m^2^) for the treatment of tubal (n = 12) and non-tubal (n = 8) EP was safe and associated with a faster time of EP resolution by 34% compared with MTX alone [145,159]. The new drug at a daily single dose of 250 mg for seven days was well tolerated by the patients with mild to moderate side effects (e.g. acne/rash and diarrhea) that are known to be associated with gefitinib [145,159]. However, gefitinib should only be used for a short and limited 7 days course and women with significant pulmonary comorbidities and Japanese ethnicity should be excluded to decrease/eliminate the risk of interstitial lung disease during the treatment of EP with gefitinib [145,159]. RCTs are still needed to confirm the aforementioned findings about the efficacy and safety of combining gefitinib with MTX for the treatment of EP [160].

#### Expectant management

When serum hCG is below the discriminatory zone and there is no intra- or extrauterine pregnancy detected by TVS, the pregnancy can be described as being PUL [121,150]. Several studies have reported that 44-69% of PUL resolve spontaneously [161,162] and 8.7–42.8% of PUL will eventually be diagnosed as early EP which were too small to visualise on initial ultrasound scan [112,114,163].

Expectant management is an option for clinically stable women with serum hCG levels below the discriminatory zone, minimal symptoms associated with either PUL or EP diagnosed on ultrasound [161,164,165]. Recent results from an RCT study showed that there is no significant difference in the outcome between MTX and expectant management groups in suitable patients [165]. Another Australian research group announced recently the initiation of a double-blinded multicentre RCT to compare between medical and expectant management for EP but the results has not been published yet [166].

Patients with EP may have an initial 50-66% increase in β-hCG concentrations every two days [112,165], mimicking a viable IUP. However the eventual fate of EP is either spontaneous resolution or rupture. This is dependent on the activity of the invading trophoblast tissue, with less aggressive invasion of the trophoblast tissue being associated with spontaneous resolution and more aggressive invasion leading to tubal rupture [167,168]. If the serum hCG concentrations increase, intervention is essential or the patients may suffer ruptured EP. On the other hand, for patients at an early stage, with lower gestational age and declining β-hCG titres, the risk of rupture is small [161,165].

Regular follow-up is essential if expectant management is to be successful and clear information about the importance of compliance with follow-up should be given to the patient. Serial serum hCG concentrations should be followed until they reach < 15 IU/L. If symptoms and signs of EP develop, serum hCG concentrations rise above discriminatory zone or start to plateau, active intervention should be considered [164,165].

#### Management of heterotopic pregnancy

The clinical management of HP aims to remove the EP without disturbing the viable IUP. Currently, there is no general consensus on the treatment of HP and the majority of data about its clinical management derive from case reports. Surgical treatment by laparotomy or laparoscopy, injection of feticides with or without fetal reduction by embryo aspiration under ultrasound guidance and expectant management have all been used and reported to be successful in the elimination of the ectopic and preservation of ongoing IUP. The selection of treatment protocol depends on the gestational age at diagnosis, the clinical condition of the patient, the site of ectopic implantation and the experience of the treating physician. In a number of studies, the success rate for rescuing the viable IUP was about 66% with the remainder ending in early or late miscarriage [108,122,169].

The treatment of HP with a tubal implantation can be performed by laparotomy or laparoscopy and the removal of EP is usually done by salpingectomy and occasionally by salpingostomy [170-174]. For interstitial HP, either medical treatment with local injection of a feticide/embryo reduction for small non-ruptured EP [158,175,176] or cornual resection by laparoscopy or laparotomy in ruptured cases has been used with rescue of the IUP [177-180]. A few studies have also reported the use of expectant management for tubal and interstitial HP [181,182].

Currently, only 14 cases of caesarean scar HP are reported in the literature. The majority of cases were treated medically by local injection of feticides and/or embryo aspiration to rescue the viable IUP except for 2 cases that were treated by laparoscopic and hysteroscopic excision of the EP masses [169,183]. Caesarean scar pregnancy can lead to massive haemorrhage due to uterine rupture and laparotomy followed by wedge excision has been reported to be the preferred approach to completely remove the EP, repair the scar and prevention of recurrence [169,184,185].

The majority of cases with cervical HP have been treated conservatively using embryo aspiration with or without local injection of a feticide to preserve the viable IUP [186-188]. Others have successfully used laparoscopy or hysteroscopy for the removal of cervical EP and preservation of the IUP [189,190]. Efficacious use of expectant management with continuous close monitoring by TVS was also reported in a few cases [141,188]. However, dilatation and curettage was also found to be necessary in some cases to prevent massive bleeding from cervical pregnancy [141,188].

Ovarian HPs have been managed by wedge resection using laparoscopy or by laparotomy in cases of massive bleeding [191]. Other cases have resulted in salpingo-oophorectomy due to massive adhesions from a ruptured EP [192]. Other ectopic sites within the abdominal cavity have also been reported and have been treated surgically due to bleeding.

#### Anti-D immunoglobulin

According to NICE guidelines, anti-D immunoglobulin at a dose of 250 IU should be given to all non-sensitized women who are rhesus negative and who have EP [139].

### Complications of ectopic/heterotopic pregnancy

#### Persistence of trophoblastic tissue

Persistent trophoblast can follow either laparoscopic or open salpingostomy with reported incidences of 8.1% and 4%, respectively [26,137,193,194]. Risk factors for persistent trophoblastic tissue include high preoperative serum hCG (>3000 IU/L), a rapid preoperative rise in serum hCG and the presence of active tubal bleeding [135,136]. Failure of serum hCG concentrations to decline following initial management is the main diagnostic sign [135,194].

There is no common protocol for the early diagnosis and initiation of treatment of persistent trophoblast [194]. Some have recommended second line treatment if the serum hCG is greater than 10% of the preoperative level ten days after surgery while others have suggested starting treatment if serum hCG concentrations are > 65% of their initial levels at 48 hours after surgery. Treatment with a single dose (50 mg/m^2^) of MTX has been widely used as an alternative to a second surgical procedure. One RCT compared the use of prophylactic MTX at the time of laparoscopic salpingostomy with simple salpingostomy alone and showed a reduction in the rate of persistent trophoblast by 19% and 14%, respectively [135].

#### Complications associated with heterotopic pregnancy

Miscarriage of the viable IUP can follow treatment of a HP. Clayton et al. reported that 84 (40.6%) out of 207 heterotopic cases ended with abortion either spontaneously or induced [195]. HP was 30% less likely to result in live-birth than an intrauterine only pregnancy following IVF treatment mainly due to the increase in risk for spontaneous and induced abortion in HP by 2 and 10 times compared with singleton IUP, respectively [195].

It is not known whether there is a difference in miscarriage rate between the different types of HP mainly due the rarity of the condition. However, early diagnosis and use of conservative approaches should lead to a better chance of live birth of the co-existing IUP. Hence, a more common site of ectopic implantation (e.g. tubal) may have a better prognosis compared with less frequent sites (e.g. caesarean scar). Future studies are needed to allow comparison of outcomes between the different sites of ectopic implantation and/or different treatment approaches, possibly using a large retrospective survey of IVF registries.

HP can result in complications including severe bleeding and hypovolemic shock, preterm delivery, uterine rupture in cases with caesarean scar implantation or oophorectomy in cases of complicated ovarian implantation [108,173,183].

### Effect of ectopic pregnancy and its treatment on subsequent IVF treatment

The effects of the different lines of treatment for EP, especially radical and medical managements, on subsequent IVF have been described. The significance of salpingectomy in the outcome of subsequent IVF-ET is controversial. It was suggested that salpingectomy could have an adverse effect on ovarian blood supply and subsequently on ovarian steroid production, further follicular development and ovarian response during later IVF cycles due to the disturbance of the vascular and neural connections between the Fallopian tube and ovary [196,197]. Many investigators reported significantly fewer follicles and oocytes either from both ovaries or the ipsilateral ovary following salpingectomy [196,198-201].

However more recent retrospective studies that have measured parameters of ovarian response in women who underwent ovulation induction before and after salpingectomy have shown no significant difference in basal FSH levels, oestrogen concentrations, length of stimulation, number of follicles, number of retrieved and fertilised oocyte and quality of embryos between the pre- and post-salpingectomy cycles [202-206].

Results about the requirement for gonadotropins for superovulation are also conflicting. While two studies showed no significant difference [202,207], another described a significant increase in the doses needed following surgery [203]. Ye et al. also reported that serum concentrations of anti-Mullerian hormone were significantly lower and FSH were significantly higher in patients with salpingectomy compared to women with no tubal surgery. However there were no significant differences in peak oestradiol concentration, endometrial thickness, number of retrieved oocytes or pregnancy rate between the study groups [207].

Surgical treatment of an EP may result in oophorectomy, either because of an advanced ovarian pregnancy or when tubal pregnancy forms a tubo-ovarian mass due to the presence of extensive pelvic adhesions [192,208]. Women with a single ovary represent a distinct treatment group when ART is needed. Many studies have shown that women who have undergone unilateral oophorectomy respond less well to ovarian stimulation than women with both ovaries, in terms of number of follicles, oestradiol concentrations and number of oocyte retrieved [208-214]. These women also seem to require a higher dose of gonadotropins in order to obtain an adequate ovarian response [208,211,214]. However, pregnancy rates in these women were similar to those with intact ovaries in the majority of these studies.

Interestingly, a very recent study has reported that despite the observed overall decrease in the number of follicles and oocytes retrieved seen in women with a single ovary, it appears that the remaining ovary is able to compensate. This was indicated by an increase in the number of follicles and oocytes obtained in patients with a single ovary compared with the response of the ipsilateral ovary of control women during IVF treatment [215]. It appears therefore, that once women with single ovary reach embryo transfer, they can be reassured that their chance of having a child is the same as for women with two ovaries.

MTX is the most accepted alternative to surgical treatment of EP and it was thought that it could potentially compromise female fertility by affecting growing follicles [216,217]. However, it appears that the cytotoxic agent does not have any harmful effect on ovarian reserve as primordial follicles in the ovaries are not affected following treatment with MTX [218-222]. Additionally, there was no significant difference in the ovarian reserve in subsequent IVF cycles between women treated with laparoscopic salpingectomy and those treated with MTX [206,222,223]. Hence, it is reassuring to show that the effects of MTX, after it is used as a medical treatment for an EP, does not affect or further compromise a woman’s future reproductive potential [206,222,223].

## Conclusions

Ectopic pregnancy is a worldwide medical emergency and its incidence increases following treatment of infertility. Heterotopic pregnancy is also more common following IVF-ET. Early diagnosis and prompt intervention are crucial in order to diminish the morbidity and mortality of EP/HP. The combination of TVS and quantitative serum hCG is currently the most reliable diagnostic tool. Considering the increase risk of EP/HP subsequent to IVF-ET, follow-up of those patients with a positive pregnancy through early pregnancy is vital and the performance of TVS between weeks 4 and 6 of gestation could allow early detection and conservative management.

Patients with PUL should be closely monitored by serial measurement of serum β-hCG and repeated ultrasonography. The finding of empty uterus by TVS and persistent increase in hCG concentration should trigger an established protocol for detection of EP. Heterotopic gestation presents a particular diagnostic challenge since the diagnostic value of hCG is limited due to the co-existing viable IUP. The detection of a HP requires a high index of suspicious coupled with TVS by an experienced operator.

Treatment approaches should be tailored according the clinical condition and future fertility requirement of the patient and they should be offered alongside with psychological support. The patient should also be provided with accurate and reliable information on risks of the different treatment approaches and their associated complications and be reassured about the preservation of prospective fertility in subsequent IVF-ET cycles.

New diagnostic tests are needed to better identify those who are at highest risk of developing EP during assisted conception. Special measures might then be adopted in order to avoid this complication, for example by elective salpingectomy or tubal clipping at the cornu as is practised for women with hydrosalpynx. Although the majority of cases can be quickly diagnosed using existing methods, diagnostic dilemmas in the form of PUL or suspected HP still present challenges to the reproductive medicine specialist. Improvement in ultrasound diagnosis of early embryonic localisation and viability, perhaps coupled with novel diagnostic tests for the presence of an extra uterine pregnancy will make the clinicians job easier in the future.
